# Impacts of sex and ethnic background on the relationship between stress and hippocampal volumes

**DOI:** 10.1002/alz.089966

**Published:** 2025-01-09

**Authors:** Katie Stypulkowski, Nikki L Kaplan, Taylor F Levine, Jessica ZK Caldwell

**Affiliations:** ^1^ Cleveland Clinic Lou Ruvo Center for Brain Health, Las Vegas, NV USA

## Abstract

**Background:**

Stress is known to have a negative impact on hippocampal volume (HCV) and convey higher risk for development of Alzheimer’s disease (AD). Previous research has established differences among older adults by racial/ethnic background in levels of stress (Brown, Mitchell, & Ailshire, 2020) and HCV (Bygrave et al., 2022). Further, women have been shown to retain brain volumes longer compared to men in the presence of other risk factors for AD (e.g., Cieri et al., 2022), but this research is primarily in non‐Hispanic White individuals (NHW). The purpose of this study was to examine the relationship between stress and HCV across a multiethnic cohort, with the hypothesis that sex would moderate the relationship.

**Methods:**

Data from the Health & Aging Brain Study ‐ Health Disparities was used, including African American (AA; *n* = 482), NHW (*n* = 817), and Mexican American (MA; *n* = 727) older adults. Prior to statistical analyses, HCVs were corrected via proportion of total intracranial volume. Six moderation regression analyses were employed to examine effects of chronic stress on HCVs. The Chronic Stress Scale total score was the independent variable, right and left HCVs were dependent variables, and sex was the moderator. Age and education were covariates.

**Results:**

There was a significant interaction between chronic stress and sex on right (*t*(711) = 2.53, *p* = .01) and left HCVs (*t*(719) = 2.57, *p* = .01) among MAs, such that MA men showed lower HCVs as chronic stress increased, while MA women showed no relationship, suggesting HCV resilience in the context of chronic stress. Sex did not moderate the relationship between stress and HCVs in NHW or AA groups.

**Conclusions:**

Sex was found to moderate the relationship between stress and HCV among MAs, but not AAs or NHWs. MA men’s HCVs decreased as chronic stress increased, while MA women’s HCVs remained robust to high stress levels. MA men may be more vulnerable to the impacts of chronic stress in older age, suggesting chronic stress may be an important modifiable risk factor for AD in this group. Further work is needed to understand the impacts of stress as a risk for AD across various demographic groups.

